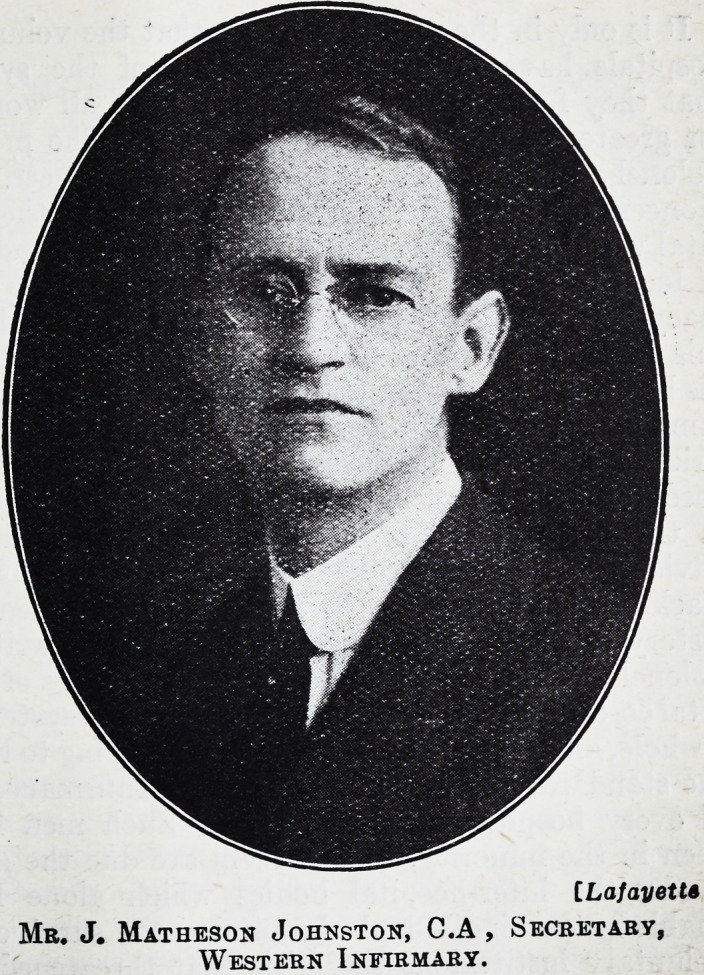# Hospital Men of Mark: Colonel D. J. Mackintosh and Mr. J. Matheson Johnston

**Published:** 1924-02

**Authors:** 


### HOSPITAL MEN OF MARK.

### Dr. D. J. MACKINTOSH and Mr. MATHESON JOHNSTON.

THE Western Infirmary, Glasgow, is an example of a great modern hospital which has grown to its present size within fifty years of its foundation. The building was begun in 1871, and was first opened for patients three years later. In 1892, when Colonel Mackintosh was first appointed Medical Superintendent, the Infirmary possessed 300 beds. Under his control it has grown to twice this size, and practically every department has been remodelled or extended. The hospitals of Scotland have generally preferred the system of placing a Medical Superintendent at the head of the administration, and among them Colonel Mackintosh is the most distinguished, for his advice is constantly sought by hospitals in all parts of the United Kingdom, especially upon questions of construction and the adaptation of buildings. Educated at Glasgow University, his first hospital posts were at the Eye and Fever hospitals of the city, and in 1890 he became Medical Superintendent of the Victoria Infirmary there. The long list of distinctions conferred on him is an index of the width of his interests in hospital and allied activities, and these range from ambulance work to the Territorials and from the College of Nursing to Health Insurance.

[Annam, Glasgow.

COL. D. J. MACKINTOSH, C.B., M.V.O., M.B., LL.D., MEDICAL SUPERINTENDENT, WESTERN INFIRMARY.

### THE MEDICAL SUPERINTENDENT'S WORK.

His book on "The Construction, Equipment and Management of a General Hospital," which was first published in 1909, and reprinted seven years later, is a valuable work on the subjects treated. During the War Colonel Mackintosh was appointed Assistant Director of Medical Services, and in 1915 became supervisor of the administration of all Military and Territorial General Hospitals in the Glasgow area. In 1917 his services were rewarded with the C.B., military division, having previously been awarded the M.V.O. in 1902. Colonel Mackintosh has long been a familiar figure at the conferences of the British Hospitals Association, and is a former Chairman of the Council. He does not pose as a public speaker, but he excels at resolving difficult questions into their elements, and at devising schemes which commend themselves to impartial opinion. It is the quality of the work that he has accomplished that has made his advice so much sought, and no one will ever be able to say that he has not earned his reputation. On matters of hospital construction his authority is recognised even in America, where he has jointly edited the series of articles upon his special theme that appear in the *Hospital World*. He would perhaps have been unable to achieve so much for so many different institutions if the Scottish system did not combine with the Medical Superintendentship a layman to devote himself to the duties of Secretary and Treasurer.

[Lafayette

MR. J. MATHESON JOHNSTON, C.A, SECRETARY, WESTERN INFIRMARY.

### VOLUNTARY HOSPITALS PROBLEM.

The qualification for the latter post is usually that of a chartered accountant, and the present Secretary and Treasurer, Mr. J. Matheson Johnston, became associated with the work of the Western Infirmary, with that qualification, in 1901. Five years later he became Assistant Secretary, in which post he remained till 1912, when he was appointed to the senior post. These two men, therefore, have served their hospital for the joint term of fifty years and worked together for twenty. Glasgow is a school of hospital finance, for the problem of maintaining the hospitals by voluntary means has nowhere proved more difficult. An important source of income is the contributions of the employees, which last year amounted to £21,114, despite the inevitable falling off that always attends a period of unemployment. Mr. Johnston has interested himself in the creation of this and other hospital funds, and since 1910 has been Assistant Secretary and then Secretary and Treasurer of the Sunday Fund movement in Glasgow.

### How it is Solved.

It is only in the past fifty years that the voluntary hospitals have grown self-conscious of the system that they form, and even to-day we are all working for greater co-ordination among them. The men of Colonel Mackintosh's generation have been the pioneers of this, and the sign of eminence in the hospital world is now recognised to be this transcendence of local interests. We recall with pleasure the intimacy between Colonel Mackintosh and the founder of this journal, to whom Colonel Mackintosh dedicated his book. The two men often worked together for the same cause, and both were ardent supporters of the British Hospitals' Association. Both urged the need for hospital men to co-operate with architects in the construction of hospital buildings, and the advice of both was frequently in request, as Colonel Mackintosh's still is at the present day. When the history of the voluntary system comes to be written, Colonel Mackintosh will be among the pioneers of hospital co-ordination, who saw the needs of the system as a whole, and spent the best of his life in trying to bring the standard, not only of the Western Infirmary, but of every hospital to a high level. Such men have been in the minority, but to them are due the great funds, the inter-hospital bodies which alone have enabled the individual hospitals to survive as voluntary institutions the crisis that threatened the system itself after the War.

### PITY THE CERTIFICATE-WRITING DOCTOR.

We are constantly being assured that the time is not far distant when medical examinations will be required not only, as now, of applicants for life insurance and would-be entrants into the Army and Navy, but also of persons requiring a license to drive a car or marry a wife. Sweden already demands medical certificates as a preliminary to marriage, and in Denmark a license to drive a car is not granted unless, among other things, a medical certificate of physical fitness is produced. There is much to be said in favour of this movement to make doctors vouch for the health of applicants for precarious tasks such as driving a motor and marrying a wife, but not infrequently the doctor is put in an invidious and even painful position, as witness the following case. An applicant in Denmark for a motor driver's license had an artificial leg. The examining doctor certified that " apart from the fact that his right leg has been replaced by an artificial limb, he is perfectly able-bodied." The applicant was refused a license, and he therefore hied him again to the same doctor for a new certificate, which was worded thus: " I certify herewith that Hr. Y. has shown himself, on examination to-day, to be in possession of the necessary mental and bodily fitness required for driving a motor." This certificate procured the applicant a license, which he held until the refusal of his earlier application travelled round to the police station where the license had been granted him. In answer to inquiries, the doctor explained that he had written the second certificate as a supplement to the first, presuming that the two would be submitted to the same authority. But this explanation did not save him from a public reprimand.

### A CRIPPLES HOSPITAL FOR PORTSMOUTH ?

It having been agreed that better provision is needed for the treatment of crippled children in Portsmouth, a public meeting has been held to decide whether an independent cripples' hospital should be established or whether a department added to the Royal Portsmouth Hospital would suffice. It is feared that the latter might suffer were a new institution established, and that the cost would also be unnecessarily great. A compromise is expected, since agreement has not yet been arrived at. The compromise would take the form of a local clinic for cripples and preliminary treatment, an orthopædic ward at the present hospital, and a cripples' home to be built at Portsdown Hill for special and convalescent cases. So much goodwill has been shown that if a generous donor should quickly come forward to give the proposal a good start, the project, which everyone admits to be necessary, would probably be realised in a short time.

### A WIRELESS MEDICAL SERVICE.

It may not be general knowledge that in certain European countries, such as Norway and Sweden, a system is already in operation which provides for wireless consultations between ships at sea and certain hospitals. It is anticipated that this system will shortly be adopted also in Denmark, and there is much to be said in its favour. It is not likely that it will render the services of a ship's medical officer superfluous ; a skipper could probably obtain little help in setting a fracture or arresting a hæmorrhage from wireless advice, however authori, tative its source. But a medical officer of a ship particularly if he be young and inexperienced, would often find it most helpful to be able to talk by wireless with his hospital teachers, and in administrative matters and questions relating to quarantine he could pick the brains of his seniors to the benefit of all concerned. The medical profession has always set its face against consultations at a distance and by post, and it is self evident that distant consultations by wireless must fall far short of perfection. But though limited, the sphere of usefulness of wireless in this connexion is great and growing.

## Figures and Tables

**Figure f1:**
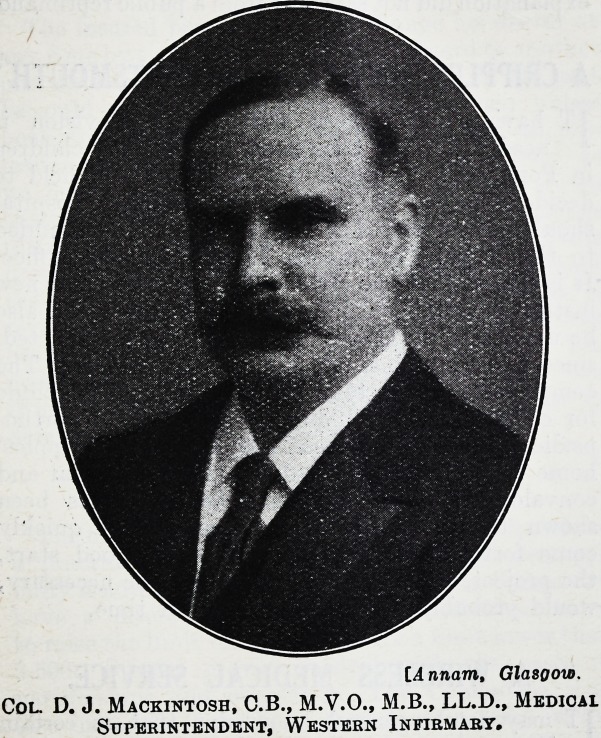


**Figure f2:**